# BSPAT: a fast online tool for DNA methylation co-occurrence pattern analysis based on high-throughput bisulfite sequencing data

**DOI:** 10.1186/s12859-015-0649-2

**Published:** 2015-07-11

**Authors:** Ke Hu, Angela H. Ting, Jing Li

**Affiliations:** 1 Department of Electrical Engineering and Computer Science, Case Western Reserve University, Cleveland, 44106 Ohio USA; 2 Genomic Medicine Institute, Lerner Research Institute, Cleveland Clinic Foundation, Cleveland, 44195 Ohio USA

**Keywords:** DNA methylation, Bisulfite sequencing analysis, Methylation co-occurrence patterns, Allele-specific methylation

## Abstract

**Background:**

Bisulfite sequencing is one of the most widely used technologies in analyzing DNA methylation patterns, which are important in understanding and characterizing the mechanism of DNA methylation and its functions in disease development. Efficient and user-friendly tools are critical in carrying out such analysis on high-throughput bisulfite sequencing data. However, existing tools are either not scalable well, or inadequate in providing visualization and other desirable functionalities.

**Results:**

In order to handle ultra large sequencing data and to provide additional functions and features, we have developed BSPAT, a fast online tool for bisulfite sequencing pattern analysis. With a user-friendly web interface, BSPAT seamlessly integrates read mapping/quality control/methylation calling with methylation pattern generation and visualization. BSPAT has the following important features: 1) instead of using multiple/pairwise sequence alignment methods, BSPAT adopts an efficient and widely used sequence mapping tool to provide fast alignment of sequence reads; 2) BSPAT summarizes and visualizes DNA methylation co-occurrence patterns at a single nucleotide level, which provide valuable information in understanding the mechanism and regulation of DNA methylation; 3) based on methylation co-occurrence patterns, BSPAT can automatically detect potential allele-specific methylation (ASM) patterns, which can greatly enhance the detection and analysis of ASM patterns; 4) by linking directly with other popular databases and tools, BSPAT allows users to perform integrative analysis of methylation patterns with other genomic features together within regions of interest.

**Conclusion:**

By utilizing a real bisulfite sequencing dataset generated from prostate cancer cell lines, we have shown that BSPAT is highly efficient. It has also reported some interesting methylation co-occurrence patterns and a potential allele-specific methylation case. In conclusion, BSPAT is an efficient and convenient tool for high-throughput bisulfite sequencing data analysis that can be broadly used.

## Background

As one type of epigenetic events, DNA methylation plays an important role in gene regulation and during normal development [1]. Abnormal DNA methylation patterns in CpG dinucleotides have been shown to be associated with human diseases such as cancer [2]. Analysis of DNA methylation patterns is of great importance in understanding the mechanism of DNA methylation and its functions during development [3].

Many technologies have been developed to systematically acquire DNA methylation information [4]. Bisulfite sequencing is one of the most popular methods, which uses bisulfite treated DNA samples to obtain single nucleotide methylation status. For example, ultra-deep bisulfite sequencing is designed to sequence a limited number of loci but with an extreme high coverage [5, 6], which makes analysis of methylation co-occurrence patterns feasible. Reduced representation bisulfite sequencing (RRBS) uses restriction enzymes to select regions of high CpG content in a genome for sequencing [7, 8]. Whole genome bisulfite sequencing (WGBS) provides an unbiased assay of methylation information across the genome [9].

Along with the generation of bisulfite sequencing data, many bisulfite sequencing data analysis tools have been proposed in recent years. Among them, QUMA [10], BISMA [11] and BiQ Analyzer [12] are earlier tools for bisulfite sequencing data analysis that have been widely adopted. However, none of the tools can handle large datasets with ultra-high read coverages or a large number of targeted regions, which are increasingly common in real data analysis. For example, QUMA web server limits the maximum number of bisulfite sequence reads per request to 400. Similarly for BISMA, the number of sequences that can be uploaded is limited to 400. The upload files size is limited to 10 MB. Even for later tools such as BiQ Analyzer HT [13] that were designed specifically for processing large datasets, their performance still cannot keep up with the throughput of data generation, mainly because they utilized a global sequence alignment algorithm. The alignment strategy also limits its usage on very small genomic regions.

More recently, some newer tools such as Bismark [14] and BS-Seeker [15] have utilized more efficient mapping tools with modifications for bisulfite sequencing data. Therefore they can effectively handle larger datasets, especially those generated by next-generation sequencing (NGS) technologies [16]. However, the primary focus of these tools is to perform sequence read map and to call methylation status at each site. Other functionalities in downstream pattern analysis and visualization are limited. Furthermore, most existing tools provide little if any functions in analyzing methylation co-occurrence patterns, nor in correlating methylation patterns with mutations. Investigating such patterns may provide further insights in distinguishing different cancer subtypes [17], in revealing mechanisms of cancer development [18], and in detecting allele-specific methylation.

In this paper, we present a web application service named BSPAT for Bisulfite Sequencing Pattern Analysis Tool, which takes advantage of Bismark’s read alignments and methylation calling functionalities, and provides further quality control, co-occurrence pattern analysis, simple allele specific methylation analysis, visualization and integration with other databases and tools. In addition to the web service, the source code of the tool is also made available, which enables advanced users to deploy BSPAT on their own machines for dedicated analysis of large volume of data without uploading them to our own server. We have applied BSPAT on a real dataset generated from two prostate cancer cell lines and one normal prostate epithelial cell line. Results have shown some interesting methylation co-occurrence patterns that are different in different cell lines. A potential allele specific methylation case is also observed. We have also compared the performance of BSPAT with a popular tool BiQ Analyzer HT [13]. Results show that BSPAT is much faster, uses less memory, and generates more results for visualization and further analysis.

## Implementation

BSPAT is designed to analyze bisulfite sequencing data for regions with extreme high read depths so that DNA methylation co-occurrence patterns can be reliably measured. It can accept reads from multiple regions and multiple experiments, which are then mapped to reference sequences by calling Bismark [14]. Based on mapping results, methylation status of a read at each CpG site is called and patterns of co-occurrence are reported. Mutations are called based on the number of reads with mismatches at each nucleotide.

### Characteristics

Comparing with existing tools, BSPAT has several important features: 1) The methylation pattern analysis features provided by most existing tools focus on either an overall methylation status of a CpG rich region or methylation level of each CpG site. Although the detailed single read methylation patterns may be presented, the significant co-occurrence patterns are not summarized. 2) BSPAT also provides a feature to automatically discover potential allele-specific DNA methylation co-occurrence patterns in a targeted region. 3) By utilizing a sequence mapping approach instead of sequence alignment algorithms, BSPAT is much faster than existing tools, as demonstrated in Result section. 4) BSPAT implements an easy to use integrated workflow and visualizes results in multiple formats.

### Workflow

**Fig. 1:**
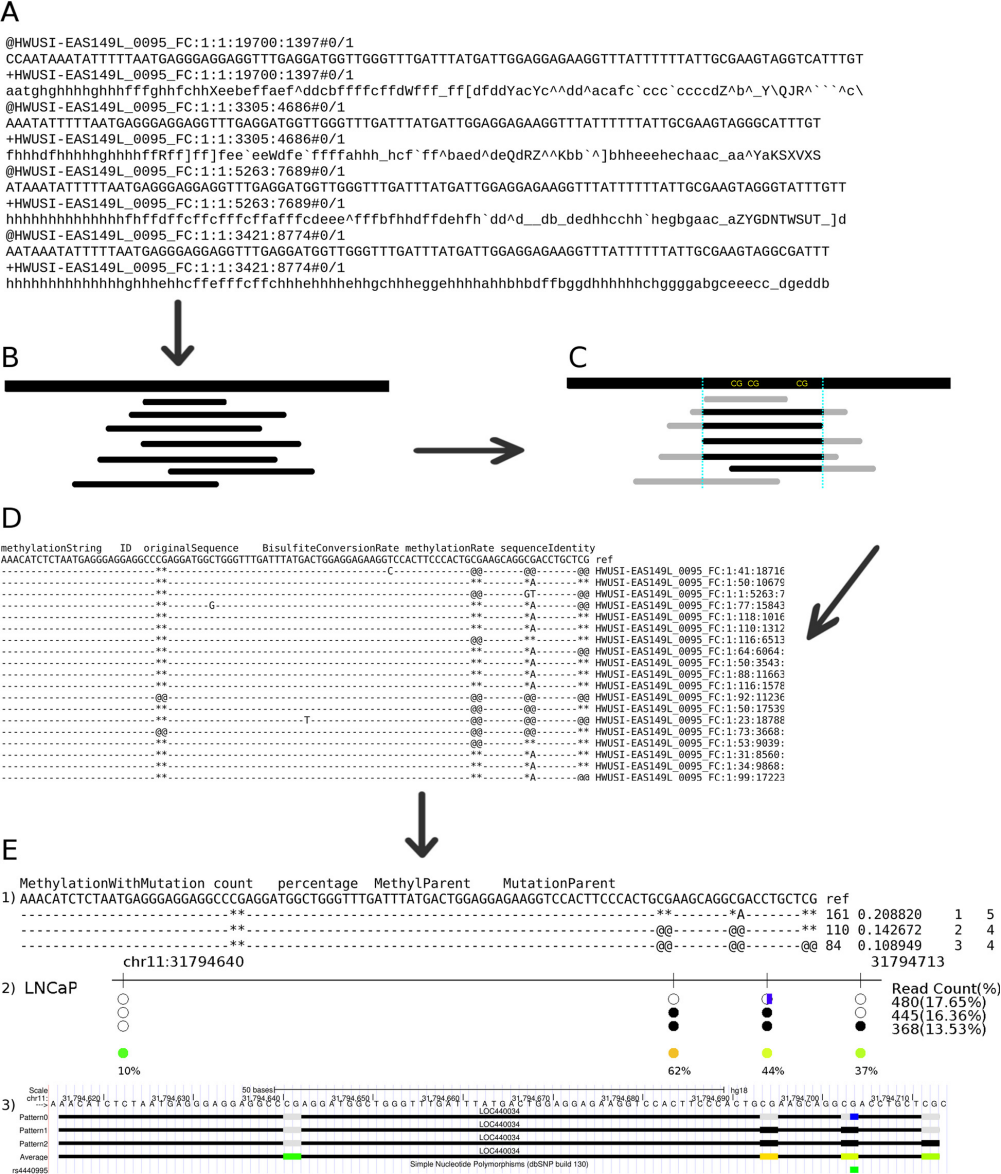
Workflow of BSPAT. **a** Example of input sequence reads in FASTQ format. **b** Sequence reads are mapped to the reference. **c** For a given targeted region, only reads that cover all CpG sites in the region are considered in generating co-occurrence patterns. d) Methylation patterns and mismatch information at single read level. **e** Visualization of results in three different formats. 1) DNA Methylation co-occurrence patterns in text format. ‘@@’ represents a methylated CpG site; ‘**’ represents an unmethylated CpG site; ‘-’ represents a non-CpG context nucleotide; a mismatch is represented by the variant allele at the position. 2) Graphical representation of methylation co-occurrence patterns with genomic coordinate information. A black circle represents a methylated CpG site and a white one represents an unmethylated CpG site. The last row represents the proportion of methylated reads to the total number of reads at each site. The colored circles show methylation rates from low (green) to high (red). Variant allele in each pattern is represented by a blue bar. 3) Methylation patterns are shown as a UCSC Genome Browser custom track

The workflow of BSPAT is shown in Fig. 1. It mainly consists of two stages: mapping stage and analysis stage. We discuss both of them in details in this subsection. For sequence reads generated from bisulfite sequencing projects, BSPAT accepts both FASTA and FASTQ format as its inputs (Fig. 1 a) for mapping. Four different types of quality scores (*i.e.*,phred33, phred64, solexa and solexa1.3) for FASTQ format are supported. Reads from multiple experiments can be uploaded at the same time. Each experiment can consist of one or more genomic regions. A utility script is also provided to extract data from multiplex experiments. BSPAT also requires users to provide a reference sequence file using FASTA format, which can consist of reference sequences from all the regions/experiments. Because the program uses a mapping strategy instead of an alignment strategy, it assumes read lengths are smaller than the lengths of reference sequences. The design of BSPAT is mainly for targeted sequencing data, where the regions sequenced are known a priori. Therefore, users should provide reference sequences of targeted regions, not the whole human genome, to speed up the mapping and analysis. To obtain genome coordinates of these regions for the analysis stage, BSPAT calls Blat service hosted by UCSC Genome Browser [19, 20] to automatically acquire the genome coordinates of reference sequences. Three versions of genome assemblies (*i.e.*, hg38, hg19, hg18) are supported currently. The top Blat result for each region, which in general represents the true region, will be selected for use in the analysis step. To map bisulfite converted sequence reads to reference regions, BSPAT relies on another program Bismark (Fig. 1 b), which actually calls Bowtie [21] to perform the mapping. The mapping step takes the majority of execution time. BSPAT allows up to three mismatches in the seed region of each read but gaps are not allowed. Reads with low mapping qualities are discarded. Users will be notified by email (if provided) when the mapping result is ready. A unique identifier is assigned to each executed job and users can use that number to retrieve the results. The webpage will also be refreshed when the result is ready, which provides some summary information about the mapping result, the genomic coordinates of the targeted regions, and a link to the detailed results generated from Bismark.

Based on mapping results, BSPAT not only summarizes the methylation level at each CpG site, more importantly, it examines methylation co-occurrence patterns of CpG sites in close proximity. BSPAT does so in several steps. First, low quality reads will be filtered out based on user-defined parameters such as bisulfite conversion rate and sequence identity. Second, in order to view co-occurrence patterns, a user needs to specify a window by providing its genomic coordinates. If no such window is given, BSPAT uses a default window of size 70 bps starting at the first CpG site of the reference sequence. Only reads that cover all the CpG sites in the view window will be considered in generating co-occurrence patterns (Fig. 1 c). For each read, the methylation status at all CpG sites covered by the read is regarded as its methylation signature or a pattern (Fig. 1 d.) Then, all reads with the same signature will be grouped into a methylation co-occurrence pattern and the number of all such reads is the support of the pattern.

Given the noisy nature of data, in general, only prevalent patterns with enough support are meaningful/significant. To filter out random patterns, users can use a simple fraction threshold (*i.e.*, the percentage of the number of reads supporting a pattern over the number of all reads). In addition, BSPAT provides a simple *Z*-score like statistic to measure the significance of a pattern. Basically, it assumes all CpG sites in the region are independently methylated with a probability of 0.5. Therefore, for *k* CpG sites in a region, there are 2^*k*^ different patterns each with equal probability of 1/2^*k*^. Any patterns with frequencies that are significantly greater than this probability are potentially important. However, the assumption may not hold in reality in the sense that the total number of reads in the region may not be sufficiently large relative to the total number of CpG sites, and methylation status of nearby CpG sites may be correlated. Therefore, instead of this probability, we actually define the baseline probability *p*_0_ as one over the number of observed patterns in the data, to better reflect dependencies among methylated CpG sites in close proximity. Assume $\hat {p}$ is the percentage of reads supporting a pattern and *n* is the total number of reads. Then one can utilize the one-sample *Z*-test for proportions to assess the significance of each pattern, with the alternative hypothesis *H*_1_: $\hat {p} > p_{0}$. The *Z*-score can be calculated based on Equation (1), where the numerate represents the difference between the observed frequency and the expected frequency, and the denominator is the estimated standard deviation under the binomial distribution. If the *p*-value corresponding to the *Z*-score is smaller than a predefined threshold, the co-occurrence pattern is treated as significant. All significant patterns will be shown in the results in the descending order of their significance.

(1) $$ Z = \frac{\hat{p} - p_{0}}{\sqrt{\frac{\hat{p}(1-\hat{p})}{n}}}  $$

In order to assess potential allele-specific methylation patterns, BSPAT first needs to discover mutations from mapping reads. In the current implementation, it simply defines a mutation as a mismatch that is supported by an excessive number of reads, using a user-defined threshold. When a mutation exists, BSPAT naturally separates all reads into two groups: reads with the reference allele and reads with the mutated allele. For each group, BSPAT assesses the methylation level at each CpG site and assigns all CpG sites into three categories based on the proportion of methylated reads covering the sites: low methylation level (≤ 20 % reads are methylated), high methylation level (≥ 80 % reads are methylated), and intermediate level (otherwise). If the two groups corresponding to the two alleles have at least one CpG site where their methylation levels are in two different categories and the actual difference of their methylation proportions is larger than 20 %, BSPAT regards the region as a potential allele specific methylation region. Then within each group, BSPAT further generates methylation co-occurrence pattens by grouping reads with the same methylation signature.

When BSPAT finishes the analysis, it visualizes significant methylation co-occurrence patterns and allele specific methylation patterns in three different formats including text format (Fig. 1e1), graph (PNG or EPS) format (Fig. 1e2), and a format that can be loaded directly to UCSC Genome Browser [22] as a custom track (Fig. 1e3). In addition, When a mutation coincides with an existing SNP in the dbSNP database [23], a link to that SNP is provided.

### Implementation details

BSPAT was developed mainly in Java/JSP and hosted in Apache Tomcat Server. To fully utilize computation resources that may be available to users, BSPAT also supports a multiple-thread mode. In this case, each experiment is executed using a separate thread, therefore it can greatly speed up the analysis. The single-thread or multiple-thread mode can be configured when users deploy the code locally. The performance improvement using multi-threads is discussed in Result section.

## Results and discussion

To test the functions and performance of BSPAT, we have performed analysis based on a real bisulfite amplicon sequencing dataset as well as a simulated dataset based on the real dataset. The real dataset consists of three prostate related cell lines (DU145, LNCaP, PrEC), each with 24 genomic regions. DU145 and LNCaP are prostate cancer cell lines. PrEC is normal prostate epithelial cell line. Genomic DNA from each cell line was bisulfite treated. The bisulfite treated DNA was PCR amplified using primers specific for the 24 regions of interest. PCR products for all 24 amplicons were pooled for each cell line and used for subsequent Illumina next-gen sequencing library construction. To enable multiplexing, a uniquely indexed adapter was used for each cell line during library preparation. The final library for each cell line was pooled together in equal molar ratios before sequencing on one lane of Illumina GAIIx. The average length of a region is about 127 bps with the total length of all regions 3020 bps. The whole dataset contains about half million reads with read length varying from 69 to 80 bps after trimming the library index and PCR primers. With default mapping parameters (maximum permitted mismatches = 2), 93.88 % reads were mapped uniquely to the reference sequences, with an average read depth of 18,886. The unmapped reads (6.12 %) were all with low quality scores or with gaps. Default parameters were used in performing pattern analysis (*e.g.*, bisulfite conversion rate 0.95, sequence identity 0.9, *p*-value 0.05 and mutation threshold 0.2). By examining the results, we have found some interesting patterns that are potentially biologically important, which will be discussed here. More thorough analysis of the dataset will be presented elsewhere.

### DNA methylation co-occurrence pattern analysis

**Fig. 2:**
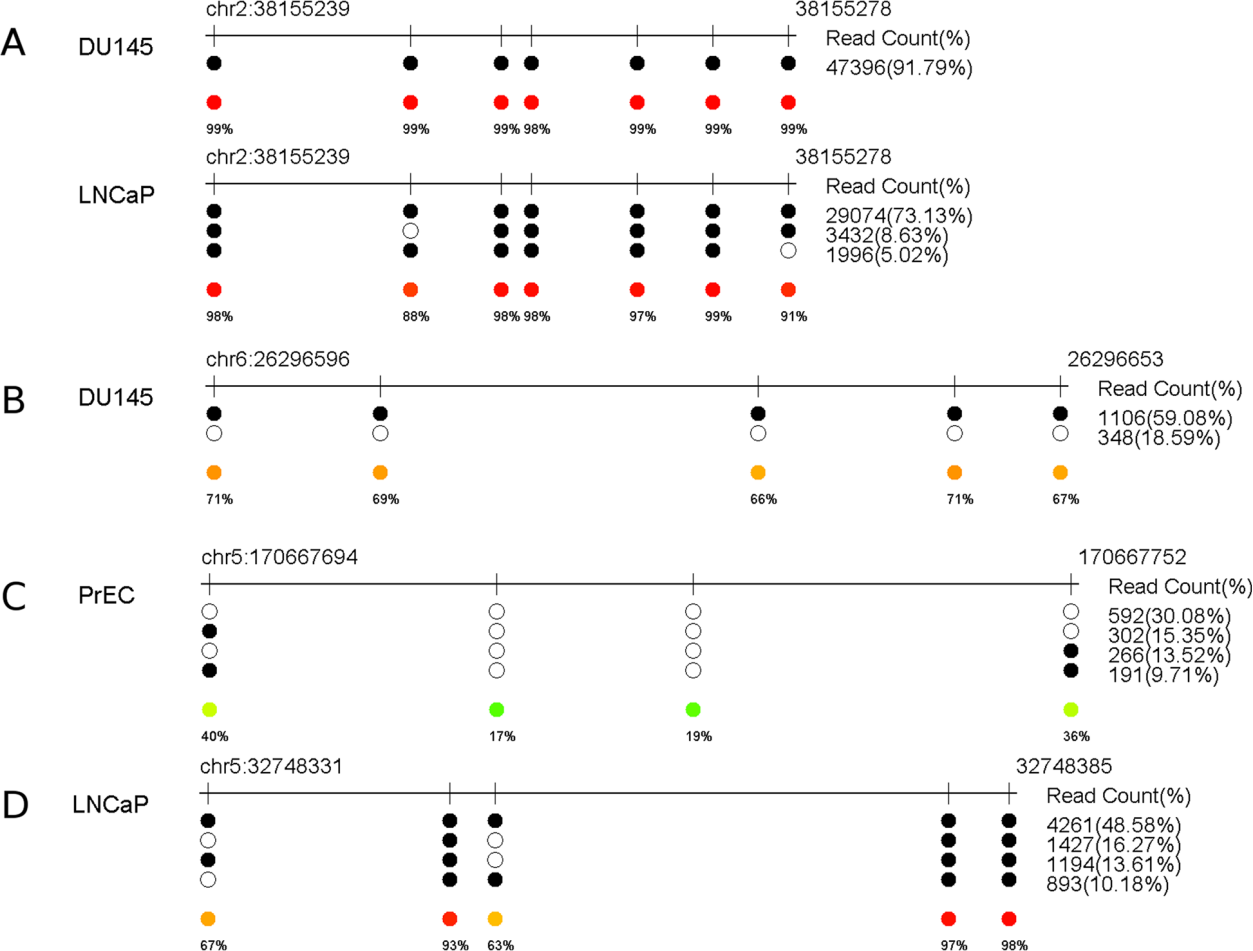
Examples of DNA methylation co-occurrence patterns. **a** DU145 and LNCaP cell lines have different significant methylation co-occurrence patterns in region *CYP1B1*. **b** Two distinct co-occurrence patterns (one all sites are methylated while the other all cites are unmethylated) in the downstream region of *HIST1H4D* of DU145 cell line. Examples of correlated partially methylated CpG sites in a region in the upstream of *TLX3* from PrEC cell line (**c**) and in the *NPR3* region from LNCaP (**d**). For all sub-panels, coordinates used are based on hg18. Because not all reads belong to a significant pattern, the sum of percentages of all significant patterns (on the right hand side of each pattern) is not necessarily 100 %

Unlike overall methylation patterns that summarize methylation levels at each individual CpG site, methylation co-occurrence patterns can reveal rich information that could be biologically important. For example, Fig. 2 a shows the methylation patterns in gene *CYP1B1* region for two cell lines DU145 and LNCaP. Although the overall methylation patterns are similar in these two cell lines, the significant methylation co-occurrence patterns are different, with DU145 showing a single significant pattern while LNCaP showing two additional patterns. The diversity may be due to the existence of sub-categories in LNCaP samples. Also, because the number of reads covering this region is extremely high, simply sorting and displaying all reads (as some other tools do) is not helpful in this case. In contrast, significant co-occurrence patterns give a clear and direct view of the methylation patterns. This is best illustrated in another example in the downstream region of gene *HIST1H4D*. There are two significant methylation co-occurrence patterns in DU145 cell line, while all CpG sites are completely methylated in one and all CpG sites are totally unmethylated in the other (Fig. 2 b). This suggests that the partially methylation status in those CpG sites are likely caused by mixture of fully methylated and unmethylated reads [24]. Some other methylation co-occurrence patterns reveal possibly correlated methylation among neighboring CpG sites. Two examples are shown in Fig. 2 c and d for genes *TLX3* and *NPR3*, respectively. For *TLX3*, methylation status of the first and the last CpG sites seems correlated, while for *NPR3*, the methylation status of the first and the third CpG sites seems correlated. By using a simple contingency table based on the read count of each pattern, we can calculate the significance level of such dependency based on a *χ* ^2^ statistics. The *p*-values for the two cases are 0.0046 and <0.0001, respectively. The observation supports the general notation that nearby CpG sites may be methylated together, but the biological mechanism of this dependence needs further investigation.

### Potential ASM detection

**Fig. 3:**
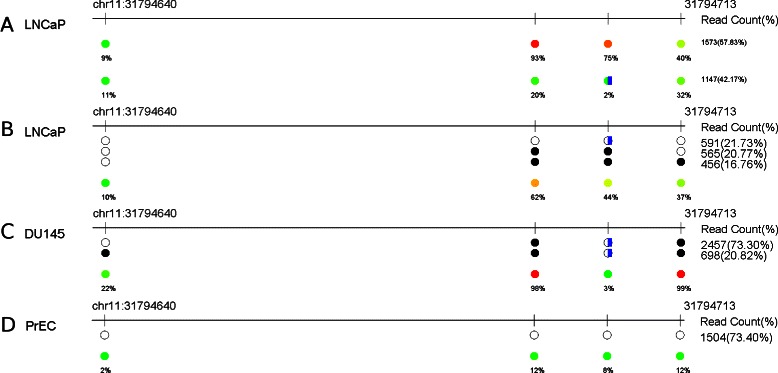
An allele specific methylation example near *PAX6*. **a** Potential allele-specific methylation patterns were discovered in LNCaP cell line near gene *PAX6*. The first row is the overall methylation level associated with the reference allele. The second row is the overall methylation level associated with the variant allele (indicated by the blue bar). Significant methylation co-occurrence patterns in LNCaP (**b**), in DU145 (**c**), and in PrEC (**d**) for the same region. Coordinates used here are based on hg18

From pattern analysis results, we have found a potential allele specific methylation pattern in *PAX6* region, as shown in Fig. 3. The mutation identified is at the third CpG site, which is also reported in dbSNP as SNP rs4440995. The nucleotide in the reference sequence is G and the variant allele is A. We first notice that in LNCaP cell line, the overall methylation levels of reads with the reference allele and reads with the variant allele are significantly different (Fig. 3 a). Further investigation based on co-occurrence patterns shows that the reference allele is associated with hypermethylation while the variant allele is associated with hypomethylation (Fig. 3 b). We further examined the mutation and co-occurrence patterns in the other two cell lines in this region (Fig. 3 c and d). Both alleles in the normal cell line (PrEC) are the reference allele while both alleles in DU145 cell line are the variant allele. The association between alleles and methylation co-occurrence patterns are different from those observed in LNCaP cell line: the variant allele in DU145 exhibits hypermethylation patterns while the reference allele in PrEC exhibits hypomethylation patterns.

There are several possibilities to explain the observation. First, PrEC is a normal cell line and has intact machinery to maintain normal methylation pattern, which is largely not methylated. This locus may be free of methylation in all normal prostate cells. In cancer cell lines, when methylation becomes abnormal, this locus gets methylated to achieve some desirable function, and the reference allele has a higher chance of becoming methylated (in LNCaP). Another possibility is the reference allele in LNCaP is in linkage disequilibrium with something that needs to be methylated here in order to achieve desirable effects. For example, the reference allele in LNCaP is linked to a wild-type protein that needs to be silenced. The SNP is linked to mutant protein already inactive. In DU145, both alleles are variant alleles and need to be silenced. Further studies and experiments are needed to confirm which hypothesis is true.

### Efficiency

To evaluate the efficiency of BSPAT on larger datasets, we have generated a simulated dataset by replicating the reads from the original data multiple times (2X, 5X, 10X, see Table 1), and compared its performance with a state-of-the-art tool called BiQ Analyzer HT. BiQ Analyzer HT is a standalone program written in Java that was developed specifically for high-throughput bisulfite sequencing data. It performs read alignments and can visualize methylation level at each CpG site and methylation status of each read. But unlike BSPAT, it does not generate methylation co-occurrence patterns. BiQ Analyzer HT can only take FASTA format input files and BSPAT can take both FASTA and FASTQ formats. We have compared the memory usage and time needed to perform the analysis by BSPAT and by BiQ Analyzer HT. All experiments were executed on the same computer with 4-core 3GHz CPU and 12 GB memory. BiQ Analyzer HT was executed in command line interface with JVM heap setting:-Xmx12g. The same JVM heap parameter was used in the Tomcat Server which hosts BSPAT. BiQ Analyzer HT can only run in the single-thread mode. We have tested BSPAT using both single-thread and multiple-thread modes (3 threads for 3 cell lines in the experiments).

**Table 1:** Sizes of datasets used in the experiments

	Read count	File size (MB)	
		FASTA	FASTQ
1X	482,791	67	134
2X	965,582	134	268
5X	2,413,955	335	670
10X	4,827,910	670	1,340

**Fig. 4:**
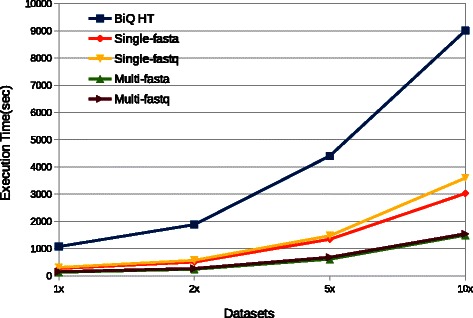
Efficiency comparison of BSPAT and BiQ Analyzer HT (referred as BiQ HT here) using different settings. BSPAT outperformed BiQ HT in all cases. BSPAT can accept FASTA or FASTQ format and run in single or multi-thread mode. All experiments were run on the same computer with quite background. For BSPAT, the Tomcat Server did not host any other applications

**Fig. 5:**
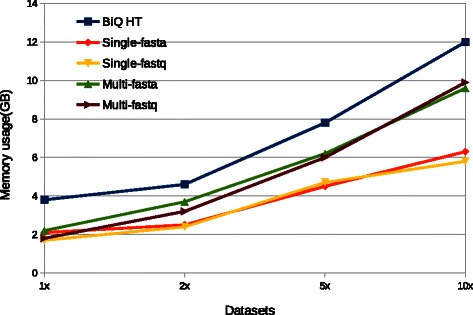
Peak memory usage comparison of BSPAT and BiQ Analyzer HT (referred as BiQ HT here) using different settings. BSPAT used less memory than BiQ HT in all cases. Here the peak memory usage of BSPAT was measured by monitoring the memory usage of Tomcat Server. For smaller datasets, the majority memory usage of BSPAT was by Tomcat Server itself. So there are no significant differences using single-thread or multiple-thread for 1X dataset

Figure 4 shows that BSPAT is much faster than BiQ Analyzer HT under all settings. When using the same setting,*i.e.*, the same FASTA format input and both using the single-thread mode, BSPAT is about 3 to 4 times faster than BiQ Analyzer HT. When using the multi-thread mode, BSPAT is about 6 to 7 times faster than BiQ Analyzer HT. The time for BSPAT using FASTQ is almost the same as the time it used for FASTA. When using BSPAT as a web service, the memory usage does not have any influence on end users. However users can deploy BSPAT in their own server. In this case, BSPAT still have less peak memory usage than BiQ Analyzer HT (Fig. 5). Comparing with BiQ Analyzer HT, single-thread BSPAT used about half of its memory. Multi-thread BSPAT utilized more memory than the single-thread version, but it was still less than the memory usage of BiQ Analyzer HT. In summary, BSPAT provides more features and has better performance than BiQ Analyzer HT both in terms of running time and memory usage.

## Conclusion

In this paper, we have presented BSPAT, a web application for methylation pattern analysis based on bisulfite sequencing data. BSPAT capitalizes on ultra deep sequence data in targeted regions to automate the n of methylation co-occurrence patterns and allele specific methylation. The implementation is efficient and also provides great flexibilities in parameter settings. Visualization of result patterns and integration with Genome Browser allow users to examine other genomic features in the same regions together. For our future work, we will refine mutation calling by combining prior information on genetic variations and more advanced variation calling algorithms. Furthermore, we will extend BSPAT to handle non-human bisulfite sequencing data.

## Availability and requirements

**Project name:** BSPAT**Project home page:** http://cbc.case.edu/BSPAT **Project source code:** https://github.com/lancelothk/BSPAT **Operating system:** Linux**Programming language:** Java**Other requirements:** Java 1.7 or higher, Tomcat 7.0 or higher, and Bismark, Perl (required by Bismark) and Bowtie (required by Bismark).**License:** GPL v3**Any restrictions to use by non-academics:** None

## Abbreviations

BSPAT: Bisulfite sequencing pattern analysis
ASM: Allele-specific methylation
RRBS: Reduced representation bisulfite sequencing
WGBS: Whole genome bisulfite sequencing
NGS: Next-generation sequencing
